# The effect of age on the early stage of face perception in depressed patients: An ERP study

**DOI:** 10.3389/fpsyt.2022.710614

**Published:** 2022-07-28

**Authors:** Hui Shi, Gang Sun, Lun Zhao

**Affiliations:** ^1^School of Foreign Languages, Qingdao University, Qingdao, China; ^2^The Department of Medical Imaging, The 960th Hospital of Joint Logistics Support Force of PLA, Jinan, China; ^3^School of Education Science, Liaocheng University, Liaocheng, China

**Keywords:** MDD, age effect, face perception, configural processing, N170

## Abstract

The aim of the present study was to investigate the age effect on face perceptual processing in MDD patients by analyzing the N170 component in response to faces and objects presented in upright and inverted conditions. For controls, although the N170 amplitude, overall, did not differ between young and middle-aged participants, the size of N170 inversion effect was larger for young than for middle-aged controls, but the N170 face effect was not influenced by age. For young participants, MDD patients showed N170 amplitude similar to controls and neither the N170 face effect nor the N170 inversion effect were influenced by depression. For middle-aged participants, MDD patients revealed larger N170 than did controls, and both the size of N170 inversion effect and the N170 face effect were larger for MDD patients than controls. These data indicate that, at least at the early stage of face perception, there is altered face perception in middle-aged but not in young MDD patients. This research could provide new evidence for clinical assessment of cognitive function in MDD patients.

## Introduction

Generally, patients with major depressive disorder (MDD) show a deficit in emotional processing, the core symptom of mental state and interpersonal behavior disorder.

Faces are important stimuli that provide basic clues related to human identity, gender, age, and emotion information, which form the basis of interpersonal communication. Therefore, people have invested a lot of energy into studying how face processing changes in MDD patients. However, most of these studies focused on the perception of facial emotion, which can convey information such as a person's mental state, intention, or personality. To date, converging evidence revealed the emotional perception deficit in MDD patients as well as its correlation with depressive symptoms [e.g., (1, 2)]. Interestingly, there was evidence that MDD patients also exhibited deficits in recognizing neutral faces (3–5). For example, when making a forced-selection response to the transient neutral, MDD patients were less able to recognize neutral faces than happy and sad faces because they could not see neutral faces as a clear sign of emotional neutrality (3). These above studies, however, focused on attention, memory, and identification of faces, rather than reflecting perceptual processing of faces *per se*. Therefore, whether depression affects the perceptual processing of faces is still controversial.

One important issue for depression is the aging effect, converging evidence showed some specific depressive symptoms in older people, that is, that late-onset depression (LOD) is strongly associated with not only aging but also disease-related brain abnormalities, with more deficits in cognitive function in comparison with younger people with depression (6, 7). However, whether there is a different cognitive dysfunction in middle-aged (especially between the ages of 40 and 55 y) vs. young MDD patients is still uncertain. Recently, based on the particularity of middle-aged people, projects on the age effects on depression have paid more and more attention to the characteristics of middle-aged MDD patients, and some valuable findings have been obtained, indicating that depression in middle-aged MDD patients is more severe than in other age groups (8). For example, there was evidence that although MDD patients overall had a significant reduced positive attentional bias and enhanced negative attentional bias, the positive attentional bias was significantly lower in MDD patients over 30 y than in young patients (<30 y), but there was no group difference in negative attentional bias. Moreover, it was found that the dosage given to middle-aged MDD patients is often larger than that given to young and elderly patients (8).

Face recognition mainly depends on configural processing, including detecting the overall relationship, first-order relationship, and second-order relationship of faces (9). Due to the interference of inversion on global/holistic face processing, the inverted face is more difficult to recognize than the upright face, that is, face inversion effect (FIE) (10). Although there was evidence that patients with depression generally pay more attention to details rather than the overall situation (11), to date, there have been no studies explore the aging effect of configural processing of faces in MDD patients. This will be investigated in the present study by analyzing the N170 of ERP (event-related potentials) components, an indicator related to the perceptual mechanism of early face processing [e.g., (12)].

As the earliest component related to facial perceptual processing, the N170 has an occipito-temporal scalp distribution, with the amplitude reliably larger for faces than other non-face stimuli (N170 face effect) and for inverted than upright faces (N170 inversion effect) [e.g., (12, 13)]. Several studies explored the early stage of facial expression processing in MDD patients by using N170 as a biomarker (14–16). For instance, when making selective responses to emotional faces, the N170 was significantly reduced and delayed in MDD patients (16). Moreover, there was evidence that, compared to the healthy controls, the N170 relevant to the emotional Go/NoGo task was significantly earlier in MDD patients, without the dominance effect of the right temporal occipital region (17).

Based on the fact that face perception is modulated by the interaction between faces' age and participants' age [i.e., own-age bias, (18)], whether there was a real altered face perception in MDD patients as well as the age effect is still uncertain. In the present study, the aging effect of processing faces would be investigated directly in young (20–29 y) and middle-aged (40–55 y) MDD patients, focusing on face detection (as reflected by N170-effect) and the application of configural processing strategies (reflected by N170 inversion effect).

## Methods

### Participants

Fifty-eight MDD patients were divided into two groups based on their age, i.e., young MDD group (30 young MDD patients: 15 females, 20–29 y, mean 25.8 ±2.2 y) and middle-aged MDD group (28 middle-aged MDD patients: 15 females, 40–55 y, mean 47.8 ± 3.6 y), and 57 age-matched healthy control participants (30 young controls: 15 females, 20–29 y, mean 26.1 ± 2.3 y, 27 middle-aged controls: 14 females, 40–55 y, mean 48.2 ± 3.5 y). In addition, there was no significant difference (*p* > 0.1) in the number of years of education (from primary school to university and graduate education) among these four groups. Each patient was recruited from the 960 Hospital of PLA (Jinan, China) and diagnosed with major depressive disorder (MDD), first episode, in accordance with DSM-V (the Diagnostic and Statistical Manual of Mental Disorders, 5th edition, Patient Edition). All patients were screened using the Structured Clinical Interview (Table 1).

**Table 1:** Demographic and clinical characteristics of major depressive disorder (MDD) and healthy controls (HC).

	**MDD**		**HC**	
	**Young** **(*****n*** = **30)**	**Middle-age** **(*****n*** = **28)**	**Young** **(*****n*** = **30)**	**Middle-age** **(*****n*** = **27)**
Gender (M/F)	15/15	15/13	15/15	14/13
Age (y)	25.8	47.8	26.1	48.2
Education (y)	13.6	14.8	14.2	14.9
Handedness (L/R)	0/30	0/28	0/30	0/27
HRSD-17	19.9	20.2	2.6	2.4
HAMA	19.8	20.1	2.6	2.5
MMSE	29.8	28.9	29.3	29.7


The severity of depression and the severity of comorbid anxiety were assessed by the 17-item Hamilton Rating Scale of Depression (HRSD-17) scores (19.9 and 20.2 for young and middle-aged patients, respectively, 1.6 and 1.4 for young and middle-aged controls, respectively, MDD vs. controls, *p* < 0.0001) and the 14-item Hamilton Anxiety Rating Scale (HAMA) scores (19.8 and 20.1 for young and middle-aged patients, respectively, 1.9 and 1.8 for young and middle-aged controls, respectively, MDD vs. controls, *p* < 0.0001), respectively. According to the Mini-Mental State Examination (MMSE) scores (29.8, 28.9, 29.3, and 29.7 for young MDD, middle-aged MDD, young controls, and middle-aged controls, respectively, *p* > 0.1), there was no patient with dementia in the major depressive disorder group. During the study period, MDD patients did not take antidepressants, mood stabilizers, antipsychotics, anxiolytics, or hypnotics (Table 2).

**Table 2:** The peak amplitudes and latency of N170 in control and MDD groups, respectively.

		**N170 peak amplitude (uV)**							
		**Upright_Face**		**Inverted_Face**		**Upright_Object**		**Inverted_Object**	
		**P7**	**P8**	**P7**	**P8**	**P7**	**P8**	**P7**	**P8**
Controls	Young	−9.5	−12.2	−11.1	−15.0	−6.3	−7.3	−5.9	−6.9
	Middle	−12.0	−10.7	−12.9	−12.2	−7.4	−7.6	−6.9	−7.3
MDD	Young	−10.4	−12.7	−12.4	−14.9	−6.2	−7.1	−5.9	−7.0
	Middle	−15.7	−15.1	−17.7	−18.4	−10.1	−9.7	−9.0	−8.9
		**N170 peak amplitude (ms)**							
		**Upright_Face**		**Inverted_Face**		**Upright_Object**		**Inverted_Object**	
		**P7**	**P8**	**P7**	**P8**	**P7**	**P8**	**P7**	**P8**
Controls	Young	157	159	161	162	160	163	162	166
	Middle	158	160	164	164	163	165	166	165
MDD	Young	157	157	163	162	162	164	165	166
	Middle	153	151	160	162	158	160	162	164

The healthy volunteers had no history of any major psychiatric disorders or major physical illnesses, and were not taking any medication known to affect the central nervous system. The exclusion criteria of the two groups also included obvious abnormal MRI results, neurological diseases, traumatic brain injury, drug use or addiction, alcohol abuse, and hearing or visual impairment. This study was approved by the Institutional Review Board of the 960 Hospital of PLA, and written informed consent of all participants was obtained before the study.

### Stimuli and procedure

The stimuli included 72 emotionally neutral faces (unfamiliar to all participants), 72 tables, and 45 butterflies, with 10.58 ^*^ 12.70 cm gray scale photographs for each. Half of the faces were male and half female, with no hair, glasses, or other accessories. There were five stimulus conditions: upright and inverted faces, upright and inverted tables, and targets (butterflies). All stimuli were presented in the center of the screen, 1.2 m away from the eyes, with the viewing angle of 5.05° ^*^ 6.06° as well as the brightness of all images equal to the root mean square (RMS) contrast (excluding the gray background in the calculation, Adobe Photoshop, www.adobe.com).

In order to effectively assign similar task associations to facial and non-facial stimuli, the passive detection paradigm was adopted in the present study, which asked participants to keep a mental count of occasionally occurring targets (butterflies) while ignoring all other stimuli. There were three blocks with 48 faces (24 upright and 24 inverted) and 48 tables (24 upright and 24 inverted) and 14, 15, or 16 butterflies (targets) for each. The stimulus trial order within each block was randomized with the inter-trial interval (ITI) of 1,200 ms as well as the stimulus presentation time of 300 ms. There was 1 min rest between each block.

### EEG recording

Continuous EEG signals were recorded at 32 electrode sites according to the 10/20 system, using a Neurolab^®^ digital EEG amplifiers (http://www.neurolab.com.cn), with a sample rate of 1,000 Hz and a band pass filter of 0.1–100 Hz as well as the tip of the nose as reference.

A common average reference was calculated off-line. After EOG artifact correction using ICA method (EEGLab software, https://sccn.ucsd.edu/eeglab/index.php), EEG signals were segmented into the epoch of 1,200 ms, including 200 ms pre-stimulus onset as the baseline correction and 1,000 ms post-stimulus onset. Epochs with an incorrect-response trial or with peak-to-peak deflection exceeding ±100 μV were excluded from averaging. The artifact-free EEG segments were separately averaged based on stimulus trial conditions. For each participant, the final averaged ERP waveforms included at least 50 trials and 30 trials for each non-target condition butterflies, respectively. For effective measurements, the averaged ERP waveforms were filtered by a low-pass filter of 30 Hz (24 dB/octave).

### Data analysis

Based on previous studies [e.g., (12)], we assessed the N170 at the lateral sites, i.e., left occipital-temporal site (LOT, P7) and right occipital-temporal site (ROT, P8). The peak amplitudes and latencies of N170 between 120 ms and 200 ms were analyzed using mixed-model ANOVA, with Depression (controls, patients) and Age (young, middle-aged) as between-subject factors, and Stimulus type (face, tables), Orientation (upright, inverted), and Hemisphere (LOT, ROT) as within-subject factors. Degrees of freedom were adjusted using the Greenhouse–Geisser epsilon correction factor for all ANOVAs and Bonferroni corrections for multiple comparisons. Planned comparisons used paired sample *t*-tests (two-tailed).

## Results

**Figure 1:**
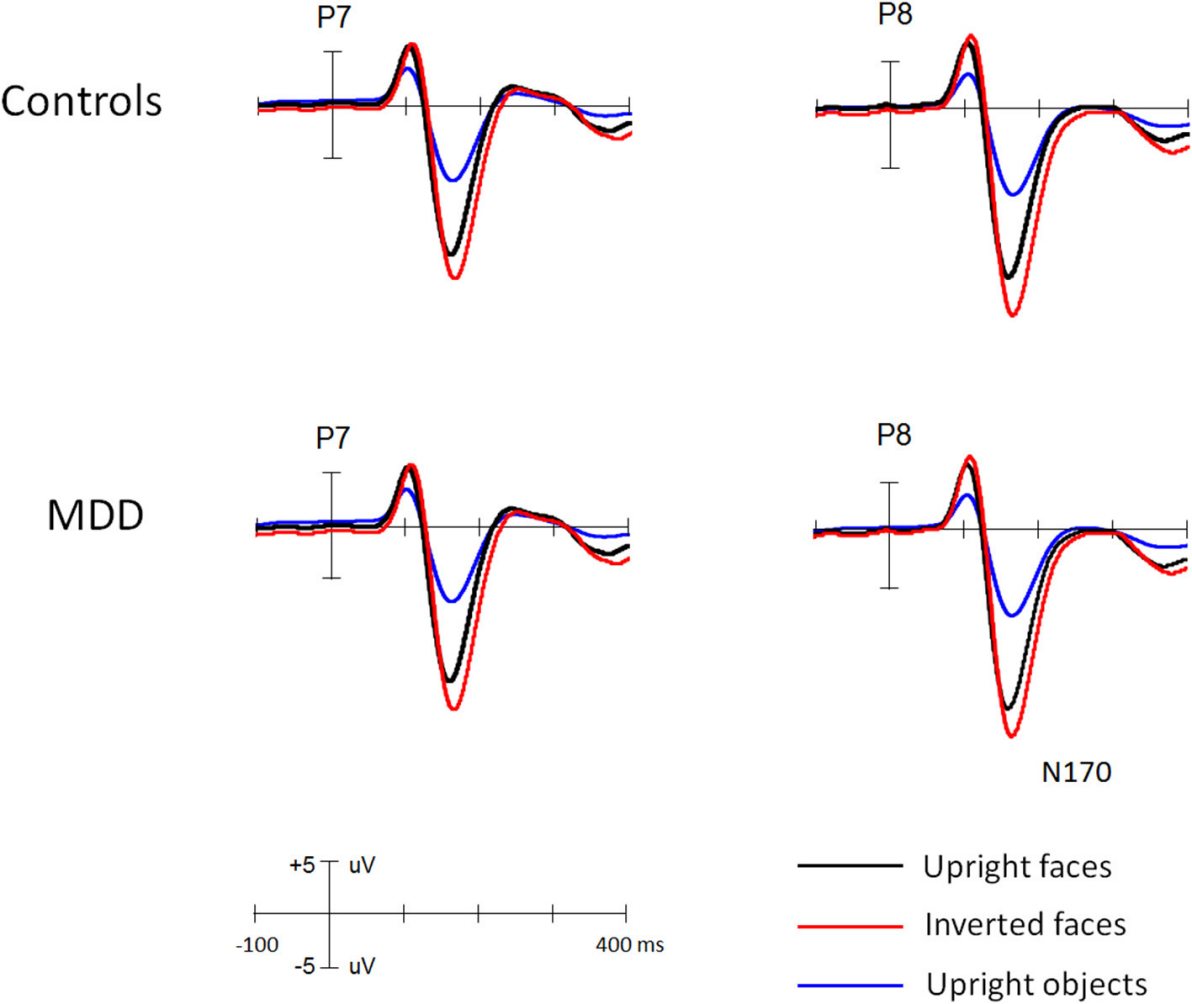
The grand ERP waveforms elicited by faces and objects in young MDD patients and controls, respectively.

**Figure 2:**
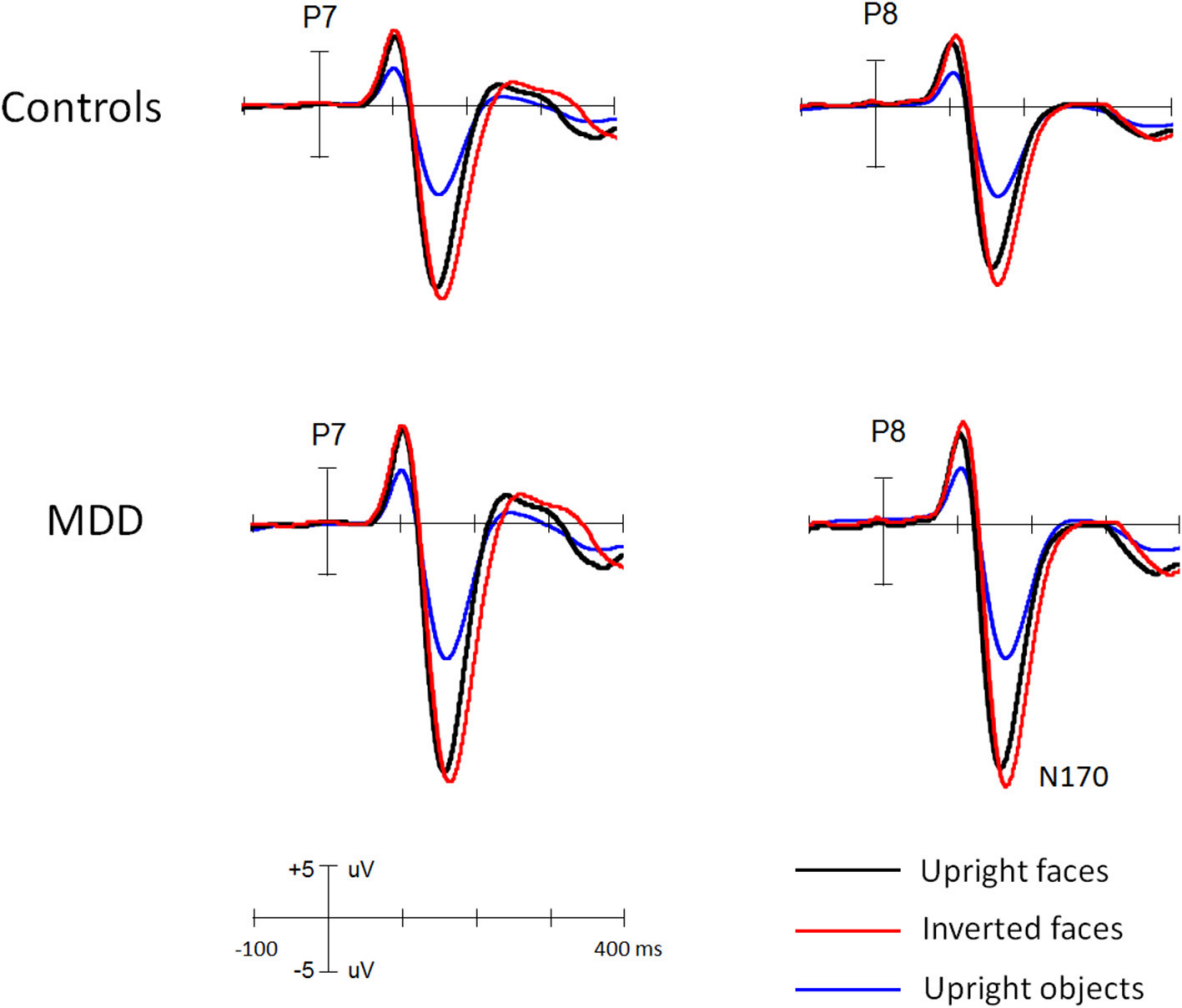
The grand ERP waveforms elicited by faces and objects in middle-aged MDD patients and controls, respectively.

**Figure 3:**
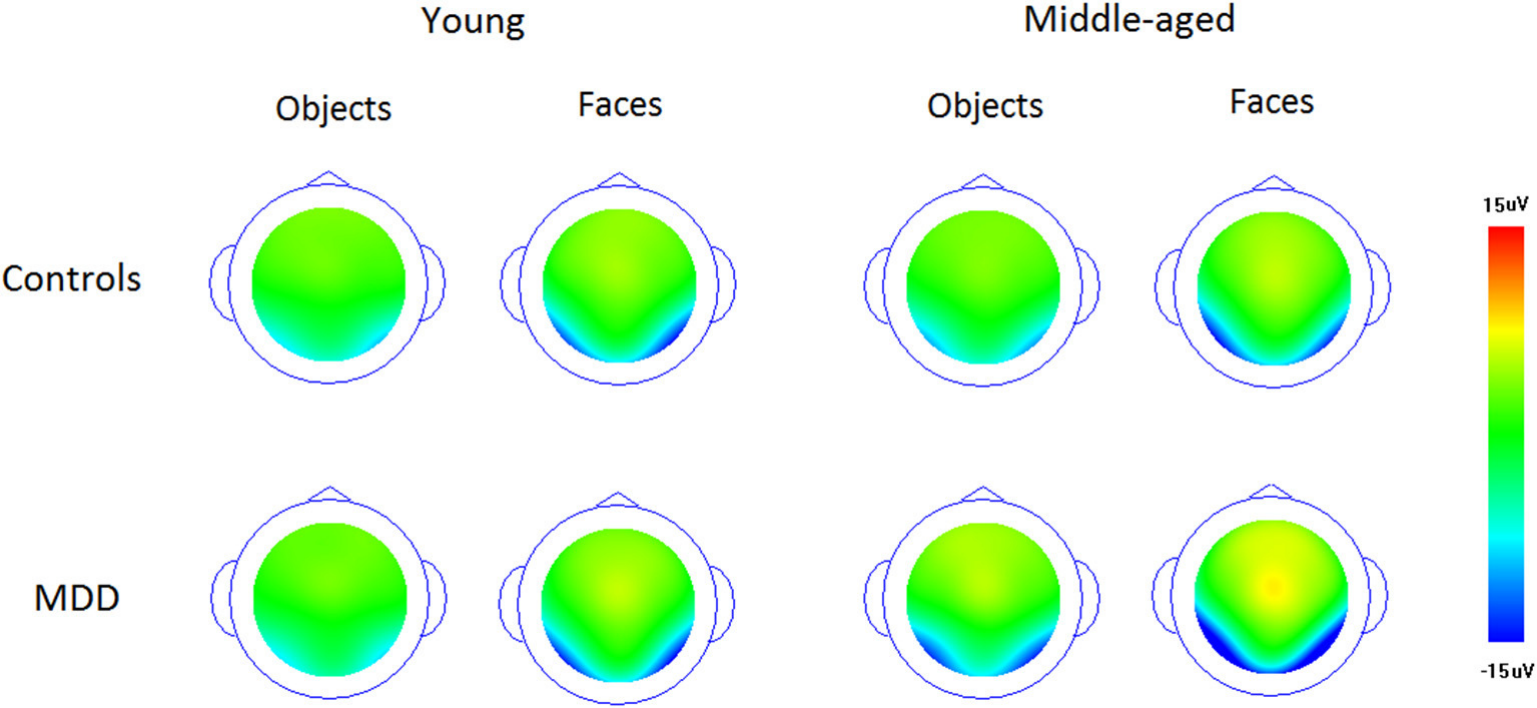
The voltage topography of N170 elicited by upright faces and objects in patients and controls, respectively.

Figures 1 and 2 showed the N170s elicited by faces and objects in different groups, respectively. Figure 3 showed the voltage topography of N170 in patients and controls, respectively. The measurements of N170 peak amplitudes and latency were presented in Table 2.

### N170 amplitude

Overall, the N170 amplitude was larger in MDD patients (−11.3 uV) than controls [−9.5 uV, *F*_(1, 111)_ = 11.9, *p* < 0.005, partial η^2^ = 0.097] groups and in middle-aged (−11.3 uV) than young groups [−9.4 uV, *F*_(1, 111)_ = 12.8, *p* < 0.005, partial η^2^ = 0.107]. Interestingly, we found the two-way interaction between two groups, *F*_(1, 111)_ = 8.8, *p* < 0.005, partial η^2^ = 0.073. *Post-hoc* comparison revealed that for young participants, the N170 did not differ between MDD and controls (9.6 and 9.3 uV for MDD and controls, respectively, *p* = 0.72), but for middle-aged participants, it was enhanced for MDD (−13.1 uV) (−9.6 uV, *p* < 0.001) and the age effect (enhanced N170 for middle-aged than for young participants) was evident in MDD participants (*p* < 0.001) not in controls (*p* = 0.663).

Across groups, all main effects were significant and qualified by two-way interaction between each other. The N170 was larger for faces (−13.3 uV) than objects [−7.5 uV, *F*_(1, 111)_ = 1195.8, *p* < 0.001, partial η^2^ = 0.915] and for inverted (−10.8 uV) than for upright [−9.9 uV, *F*_(1, 111)_ = 152.7, *p* < 0.001, partial η^2^ = 0.579] conditions. The two-way interaction of Stimulus ^*^ Orientation was significant, *F*_(1, 111)_ = 235.4, *p* < 0.001, partial η^2^ = 0.68, showing N170 inversion effect for faces (−2.1 uV, *p* < 0.001) but not for objects (−0.5 uV, *p* > 0.1).

Importantly, several interactions were qualified by two group factors. Both the Orientation ^*^ Depression [*F*_(1, 111)_ = 4.1, *p* < 0.05, partial η^2^ = 0.04] and Orientation ^*^ Age interactions [*F*_(1, 111)_ = 4.6, *p* < 0.05, partial η^2^ = 0.04] were significant, showing that N170 inversion effect was slightly larger in MDD (N170_inverted minus N170_upright: −0.8 uV) than in controls (−0.6 uV, *p* = 0.07) and in young (−0.9 uV) than middle-aged participants (−0.6 uV, *p* = 0.08). The Stimulus ^*^ Depression interaction was also significant [*F*_(1, 111)_ = 23.9, *p* < 0.001, partial η^2^ = 0.18], but qualified by three-way interactions of Stimulus ^*^ Orientation ^*^ Depression [*F*_(1, 111)_ = 7.2, *p* < 0.01, partial η^2^ = 0.06] and Stimulus ^*^ Depression ^*^ Age [*F*_(1, 111)_ = 8.3, *p* < 0.01, partial η^2^ = 0.07]. We also found significant four-way interaction of Orientation ^*^ Stimulus ^*^ Depression ^*^ Age [*F*_(1, 111)_ = 8.3, *p* < 0.01, partial η^2^ = 0.07] and five-way interaction of Stimulus ^*^ Orientation ^*^ Stimulus ^*^ Depression ^*^ Age [*F*_(1, 111)_ = 7.1, *p* < 0.01, partial η^2^ = 0.06].^1^

### N170 latency

The ANOVA analysis showed that the N170 peak latency was similar among four groups (161, 163, 162, and 159 ms) for young and middle-aged controls and young and middle-aged MDD patients, *F*_(1, 111)_ = 3.3, *p* = 0.07, partial η^2^ = 0.029). Across groups, all main effects were significant. The N170 peaked faster for faces (159 ms) than objects [163 ms, *F*_(1, 111)_ = 67.3, *p* < 0.001, partial η^2^ = 0.378], longer for inverted (163 ms) than for upright [159 ms, *F*_(1, 111)_ = 306.3, *p* < 0.001, partial η^2^ = 0.734] conditions, and at ROT (162 ms) than at LOT [160 ms, *F*_(1, 111)_ = 6.0, *p* < 0.02, partial η^2^ = 0.051] sites. Both the two-way interactions of Orientation ^*^ Depression [*F*_(1, 111)_ = 19.2, *p* < 0.001, partial η^2^ = 0.148] and Orientation ^*^ Age [*F*_(1, 111)_ = 6.9, *p* < 0.01, partial η^2^ = 0.058] were significant, and revealed a larger inversion effect of N170 latency for MDD (N170_inverted *minus* N170_upright: 5.4 ms) than control (3.3 ms, *p* < 0.05) participants and for middle-aged (5.0 ms) than young (3.7 ms, *p* < 0.05) participants. The Stimulus ^*^ Orientation interaction was significant, *F*_(1, 111)_ = 47.8, *p* < 0.001, partial η^2^ = 0.301, but interacted by Age, *F*_(1, 111)_ = 5.91, *p* < 0.02, partial η^2^ = 0.051, showing that the above group effects of the N170 inversion effect was face-specific (*p*s <0.001), but did not apply to objects (*p*s > 0.1). No other effects reached a significant level (*p*s > 0.1).

## Discussion

In order to investigate the age effect on the perceptual computation in early stages of processing faces in MDD patients, the N170 component was analyzed in response to faces and objects presented in upright and inverted conditions. For controls, although the N170 amplitude, overall, did not differ between young and middle-aged participants, the size of N170 inversion effect was larger for young than for middle-aged controls, but the N170 face effect was not influenced by age. For young MDD patients, the N170 was similar to controls and neither the N170 face effect nor the N170 inversion effect were influenced by depression. However, the middle-aged MDD patients revealed larger N170 than did controls, and both the size of N170 face effect and the N170 inversion effect were also larger for MDD patients than controls.

Inconsistent with a previous study that showed the N170 elicited by upright faces was more enhanced for the elderly (>60 y) than the younger patients (20–30 y), the N170 did not differ between young and middle-aged people, indicating that the enhanced N170 could be specific to the elderly and related to the decreased neuronal adaptation [e.g., (19)]. Importantly, in line with recent findings of the N170 in the elderly, the present middle-aged people also showed the reduced N170 inversion effect as well as the similar N170 face effect, indicating that the face processing at a basic category level (faces vs. non-face objects) could be intact for adults regardless of age, but the application of configural computations by default could be reduced with age (including the middle-aged and older people), which could be the cause of cognitive aging [e.g., (19–21)]. It could be considered therefore that, compared with young people, although there is no decline in neural refractory (reflecting by N170 amplitudes) and face perception at the basic level (reflecting by N170 face effects), the face perceptual ability (reflecting by N170 inversion effects) has actually appeared aging tendency in the middle-aged people.

For young MDD patients, we did not find any modulation of depression on the N170, regardless of N170 face effect or N170 inversion effect. Although there was evidence that MDD patients exhibited smaller N170 to emotional faces [e.g., (22)] and recognized neutral faces less accurately and more slowly (3), the present findings about the perceptual mechanism of face processing vs. non-face objects indicate that young MDD patients exhibit similar patterns (faces vs. non-face objects) to healthy controls in the early perceptual stage of face processing. It is also generally believed that face inversion disrupts configural processing of faces (23–25). Hence, the similar N170 inversion effect implies that young MDD patients could have an intact perceptual mechanism of processing faces, that is, primarily relying on global/configural information vs. non-face objects.

For middle-aged MDD patients, the larger N170 amplitude as well as the face and inversion effects of N170 is consistent with the relationship between acute psychosis and healthy participants reported by Valkonen-Korhonen et al. (26). However, in order to obtain the specificity of depression on facial processing, we included a non-facial condition and calculated the N170 face effect, indicating that the enhancement of N170 is specific to face processing rather than a more general property. The increase in amplitude of early ERP components (e.g., N170) can be explained by the decrease in neural adaptation associated with psychiatric characteristics (26). In fact, neural plasticity is the basic fundamental mechanism of neuronal adaptation, which is disrupted in depression (27). This study further confirmed that the age of MDD patients indeed modulated the decline of neuronal adaptation. Compared with controls, on the other hand, the larger N170 in the middle-aged MDD patients could also reflect a hyper vigilance to salient cues such as faces in the present study. For example, high pain vigilant subjects showed generally enhanced early (early posterior negativity) and late (late positive complex) ERP components related to processing of emotional faces, considering that in pain vigilant subjects had enhanced attentional capture particularly driven by emotional faces (28). However, age-related decline in the ability to utilize alerting cues is widely reported [e.g., (29)], and, interestingly, one recent study found that, although the elderly benefit less from visual alerting cues than the young, they can also make good use of auditory alerting cues, indicating that the aging effects of alerting mainly come from cognitive rather than neuromodulatory changes (30). In a word, the precise relationship between the hyper vigilance for salient cues (accounting for the enhanced N170) and middle-aged MDD patients remains to be determined.

In addition to N170 face effect, the enhanced N170 inversion effect for middle-aged MDD patients vs. middle-aged controls suggests that, although middle-aged MDD patients cannot sufficiently detect a face at the basic category level of faces vs. non-face objects in the early stage of perceptual processing, they may have reliable high-level visual perception relevant to the configural processing of faces, at least at the early stage of face processing. Integrating the present findings of the similar N170 inversion effect among young healthy controls, young MDD, and middle-aged MDD patients, it could be concluded that the global/configural computation of faces is not impaired by depression, regardless of the age of patients. However, there was evidence that MDD patients tend to pay attention to details and individual information, rather than the global situation, which is correlated to depression symptoms [e.g., (11, 31)]. Actually, the face inversion involves many aspects of face processing such as first-order and second-order configural processes as well as featural processing (9, 23, 24). It is necessary therefore to further verify the configural processing using the detailed paradigm in MDD patients.

Before concluding, it should be noted that, although the present findings are reliable based on strong statistics analysis, future research should expand the sample size to a larger age range including 20–29 y, 30–39 y, 40–55 y, and elderly participants and explore the applicability of this effect, especially the gender effects in MDD patients (15). In addition, the present MDD patients actually had comorbid anxiety by the 14-item HAMA scores. Although anxiety disorder could modulate the N170 in response to emotional faces [e.g., (32, 33)], there was no clear evidence for the dysfunction of face perception in anxiety disorders. It is necessary to further investigate face perception (featural and configural processing) in MDD patients without any comorbid psychiatric disorders.

In sum, the present study first investigated the age effect on face perceptual processing in MDD patients by analyzing the N170 of ERP components in response to faces and objects presented in upright and inverted conditions. Young MDD patients showed N170 amplitude similar to controls and neither the N170 face effect nor the N170 inversion effect were influenced by depression, however, middle-aged MDD patients revealed larger N170 than did controls, and both the size of N170 inversion effect and the N170 face effect were larger for MDD patients than controls. These data indicate that at least at the early stage of face perception, an altered face perception was evident in middle-aged but not in young MDD patients. The study also provides new evidence for clinical assessment of cognitive function in MDD patients.

## Data availability statement

The raw data supporting the conclusions of this article will be made available by the authors, without undue reservation.

## Ethics statement

The studies involving human participants were reviewed and approved by PLA 960th Hospital. The patients/participants provided their written informed consent to participate in this study.

## Author contributions

HS completed data collection and the draft. GS and LZ completed design, data analysis, and revised the manuscript. All authors contributed to the article and approved the submitted version.

## Funding

This work was supported by the Basic Research Key Program, Defence Advanced Research Projects of PLA (2019-JCQ-ZNM-02).

## Conflict of interest

The authors declare that the research was conducted in the absence of any commercial or financial relationships that could be construed as a potential conflict of interest.

## Publisher's note

All claims expressed in this article are solely those of the authors and do not necessarily represent those of their affiliated organizations, or those of the publisher, the editors and the reviewers. Any product that may be evaluated in this article, or claim that may be made by its manufacturer, is not guaranteed or endorsed by the publisher.

## Footnotes

^1^Based on the present aim, further analysis of the five-way interaction was conducted by separate four-way ANOVAs: the 4-way interactions of Depression ^*^ Stimulus ^*^ Orientation ^*^ Hemisphere for two age groups and Age ^*^ Stimulus ^*^ Orientation ^*^ Hemisphere for MDD and control groups, respectively. The results were presented in the supplementary section.
